# A systematic review and meta-analysis comparing suprapatellar versus infrapatellar approach intramedullary nailing for tibal shaft fractures

**DOI:** 10.1007/s00068-023-02384-9

**Published:** 2023-11-21

**Authors:** Zhongqing Wang, Xianmei Xiong, Zesheng Lu, Yijia Gao

**Affiliations:** 1 The First Clinical School of Guangzhou, University of Chinese Medicine, Guangzhou, China; 2 The First Affiliated Hospital, Guangzhou University of Chinese Medicine, Guangzhou, China

**Keywords:** Suprapatellar, Infrapatellar, Intramedullary nailing, Tibal shaft fractures, Approach

## Abstract

**Background:**

The application of the suprapatellar (SP) approach has challenged the traditional infrapatellar (IP) approach in the surgery treatment of tibial shaft fractures, yet the advantages and disadvantages still remain controversial. We included more high-quality studies for this meta-analysis and systematic review to evaluate the clinical outcomes and prognosis of both approaches and thus to provide new ideas for surgeons.

**Method:**

We searched literatures from PubMed, Cochrane Library, Web of Science, and EMBASE databases from January 2000 to December 2022. We extracted general information including sample size, gender, proportion of open fracture, follow-up time, and outcome indicators including entrance accuracy, fluoroscopy time, operation time, intraoperative blood loss, Lysholm score, VAS pain score, range of motion (ROM) function score, reposition accuracy, and revision cases. Cochrane Collaboration's tool and the Newcastle–Ottawa Scale were used to evaluate literature qualities. Meta-analysis was performed using RevMan 5.4 software.

**Results:**

A total of 23 studies were generated that qualified for inclusion, 17 of which were used for meta-analysis. This study found statistically significant differences in coronal plane entrance accuracy, fluoroscopy time, Lysholm score, and VAS pain score.

**Conclusion:**

The results of our meta-analysis showed that the SP approach was significantly better than the IP approach in angle and distance entrance accuracy of coronal plane, angle entrance accuracy of sagittal plane, fluoroscopy time, Lysholm score, and VAS pain score. There were no significant differences in sagittal angle accuracy, operative time, intraoperative blood loss, and ROM score.

## Introduction

Among all long bone fractures, the tibial fracture is the most common and accounts for the largest proportion of adolescents, of which approximately 24% are open fractures, with the fracture form often presenting as transverse oblique and spiral fractures. Tibial shaft fractures account for approximately 1.9% of all fractures in the body, with an overall incidence of approximately 20/100,000/year [1, 2]. Due to the specificity of the site, non-surgical treatment often results in poor alignment, fracture displacement, prolonged immobilization, ankle stiffness, and lower limb function [41]. Currently, the main surgical treatment measures for tibial shaft fractures are intramedullary nailing (IMN) and open reduction internal fixation (ORIF) with locking plates. However, after ORIF, complications such as surgical site infection [45], effusion, and osteomyelitis often occur [3], with a consequent risk of readmission [28], while IMN is widely used because of convenient access, less trauma, less stripping of periosteal tissue, and easy recovery with half or even full weight bearing [3].

The traditional incision point for IMN was the IP approach, separating the patellar tendon and inserting IMN from the parapatellar position. Although the minimal incision facilitated postoperative recovery, it was still challenged by the high incidence of postoperative complications such as anterior knee pain [4].In 1996, P Tornetta 3rd was the first to report an alternative surgical approach [5], the SP approach, which has the advantage that the target leg is placed in a semi-extended position of the knee, eliminating quadriceps stretch forces on the proximal fragment and valgus deformity. Intraoperatively, the procedure was performed under the condition that the knee was flexed at approximately 20 degrees. A 3 cm long incision was made approximately 3–5 cm wide above the patella to longitudinally separate the quadriceps tendon, the patellofemoral joint was further bluntly dissected, and then a trocar system was inserted through the joint space to create a starting point at the junction of the proximal tibial anterior cortex and articular surface. Intraoperative fluoroscopy as well as fracture reposition was easier to obtain due to the specific flexion position, and another potential advantage may be improving in anterior knee pain, as the new approach was unlikely to stimulate the patellar tendon or injure intra-articular structures if surgeons performed properly [40, 44]. Studies [6–8] showed that the ideal inserting point of the safety zone was located at the anterior edge of the tibial plateau and medial to the lateral tibial spine. The aforementioned safety zone was small and the intra-articular structures of the knee may be damaged if the correct point of entrance was lost.

To further investigate whether there is a significant difference in the effect of the two approaches on the surgical procedure and postoperative recovery, we compiled more and newer literature, and then performed this meta-analysis to obtain the final results. We hypothesized that the SP approach would be superior to the IP approach in terms of entrance accuracy, fluoroscopy time, functional scores, and anterior knee pain.

## Methods

### Search method

This meta-analysis was performed following the Preferred Reporting Items for Systematic Reviews and Meta-Analyses (PRISMA) statement [16]. We searched the following databases: PubMed, EMBASE, Cocharen, and Web of Science, from January 2000 to December 2022. The following keywords were entered: “Suprapatellar and infrapatellar”, “intramedullary nail”, and “tibal” “fracture” in the process. The language was set as English, without geographical restrictions.

### Criteria

Inclusion criteria: (1) adult patients with normal skeletal development and tibial intramedullary nailing for the first time; (2) RCTs or observational studies; (3) intervention controls: suprapatellar versus infrapatellar groups; (4) record of the results: entrance accuracy (include degree and distance), fluoroscopy time, operation time, intraoperative blood loss, Lysholm score (which was first published in 1982 in the form of a scale containing eight items on knee function and symptoms describing activities of daily living for patients, and is now widely used in all types of knee fractures, 6 months postoperatively), VAS pain score (6 months postoperative) and ROM scores (6 months postoperative). The exclusion criteria were as follows: biomechanical experiments, animal experiments, cadaveric studies, conference abstracts, case reports, review and meta-analyses or pathologic fractures.

### Statistics

The extracted information included: (1) research background, first author, year of publication, country, study design, sample size, average sample age, gender, proportion of open fracture, and follow-up time; (2) study results, including entry point accuracy, fluoroscopy time, operation time, intraoperative blood loss, Lysholm score, VAS pain score, and ROM function score.

### Research quality assessment

We assessed the risk of bias by using Revman for the included study type of RCT by Cochrane criteria, including: random sequence generation, allocation concealment, participant and personnel blinding, blind method of result assessment, incomplete outcome data, selective reporting, and other source bias, and evaluated them with low risk, high risk, or unclear risk. For observational studies, we used the Newcastle–Ottawa Scale for a systematic star rating, dividing the studies into a 0–9 level, with levels greater than 6 being considered as high-quality studies. Any of the above assessment differences were resolved by the co-authors through consultation.

### Statistical analysis

For the included data, all were analyzed by the Revman 5.4 software. Mean difference and 95% confidence intervals were calculated. I^2^ was used for the heterogeneity testing. A random effect model was used regardless of I^2^. Sources of heterogeneity were explored by sensitivity analysis or subgroup analysis.

## Results

### Study search

**Fig. 1:**
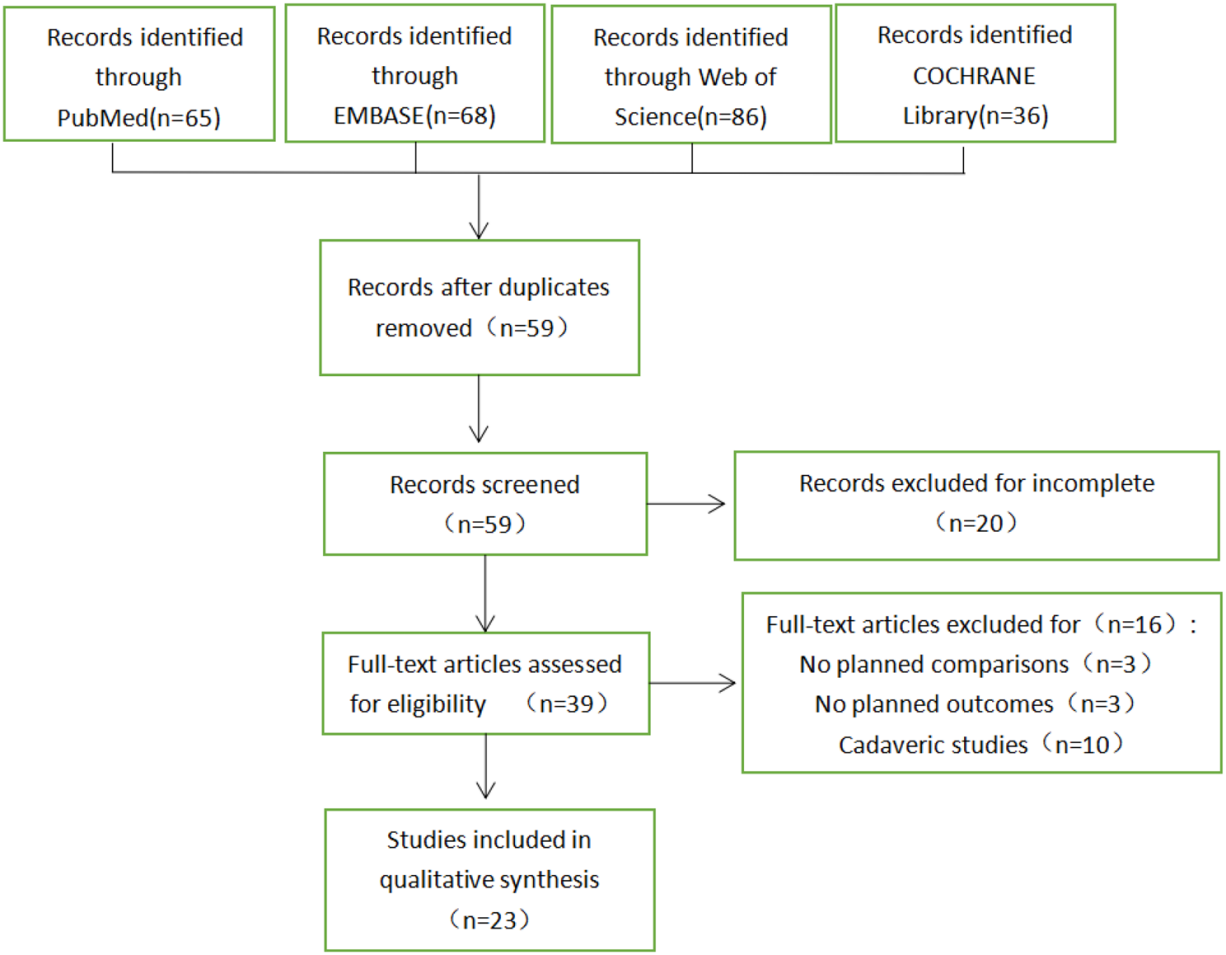
Flow diagram of the article selection process

Following the search strategy described above, 525 studies were initially screened from the four databases, and 59 were retained after removing the duplicates. By reading the titles and abstracts, 20 studies were excluded for not meeting our requirements, leaving 39 articles for the full text reading. Finally, 23 articles were selected for the meta-analysis. The searching process is shown in Fig. 1. Demographic characteristics are summarized in Table 1.

**Table 1:** Summary of study and patient characteristics

			Results of meta-analysis		
Outcome indicators	Subgroup	Number of comparison studies	MD [95% CI]	P value	I^2^ (%)
Coronal degree (°)	–	5 [20, 21, 41–43]	– 1.74 [ – 2.22, – 1.26]	< 0.00001	84%
Sagittal degree (°)	–	5 [20, 21, 41–43]	– 1.18 [ – 2.59, 0.24]	0.10	98%
Coronal distance (mm)		4 [25, 29, 33, 43]	– 1.47 [ – 2.18, – 0.77]	< 0.04	65%
Sagittal distance(mm)	–	4 [25, 29, 33, 43]	– 3.12 [ – 5.54, – 0.69]	0.01	91%
Fluoroscopy time (s)	–	4 [21, 26, 27, 43]	– 39.23 [ – 45.55, – 32.91]	< 0.00001	0%
Operative time (min)	–	8 [21, 22, 26, 29, 32, 38, 40, 41]	– 5.62 [ – 11.88, 0.63]	0.08	93%
Intraoperative blood loss (ml)	–	5 [22, 26, 32, 40, 43]	– 0.12 [ – 0.95, 1.71]	0.9	0%
Lysholm knee score	RCT	3 [24, 26, 28]	7.88 [5.52, 10.24]	< 0.00001	0%
	Observational	8 [22, 29, 31, 33, 38, 40, 41, 43]	5.05 [1.91, 8.18]	0.002	81%
	Total	11 [22, 24, 26, 28, 29, 31, 33, 38, 40, 41, 43]	5.88 [3.39, 8.36]	< 0.00001	76%
VAS pain score	–	6 [26, 28, 31, 39, 41, 43]	– 0.64 [ – 1.10, – 0.17]	0.007	96%
Range of motion	–	6 [26, 31, 32, 38, 40, 43]	3.21 [ – 1.37, 7.78]	0.17	96%

*CN* China;*UK* the United Kingdom

### Data included

3 RCTs and 20 observational studies were included following the above search strategy, and all included studies were completed by December 2022. A total of 2140 patients with tibial fractures were considered, including 1008 SP intramedullary nailing and 1132 IP intramedullary nailing therapy. Follow-up time for each study varied from 6 to 63 months.

### Risk bias and quality assessment

**Fig. 2:**
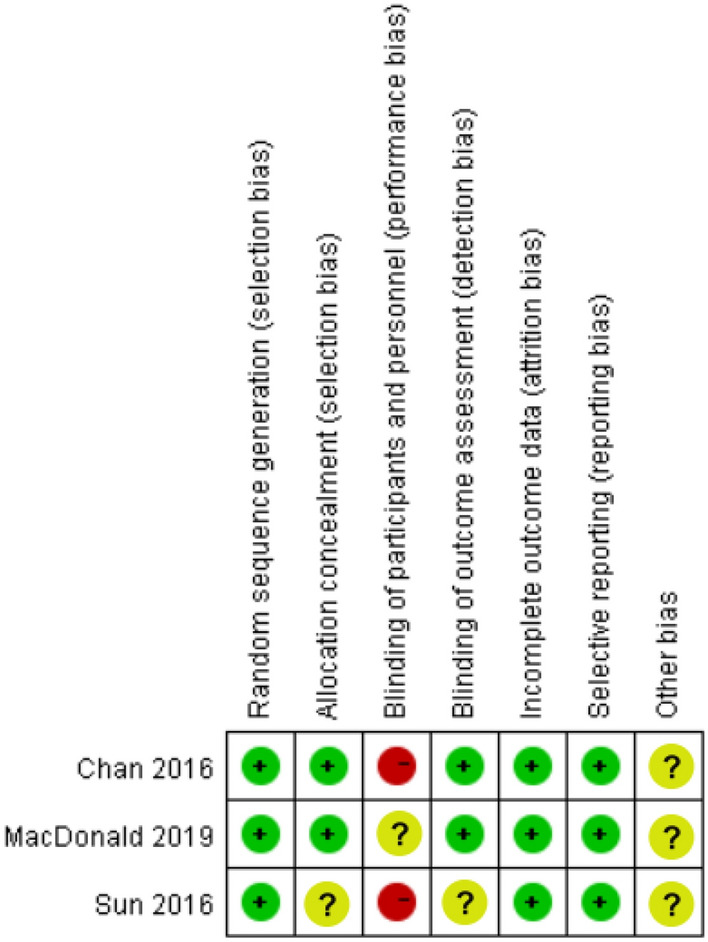
Risk of bias assessment summary of three RCTs (Cochrane tool RoB 2). Green, low risk of bias; red, high risk of bias; yellow, moderate risk of bias

**Fig. 3:**
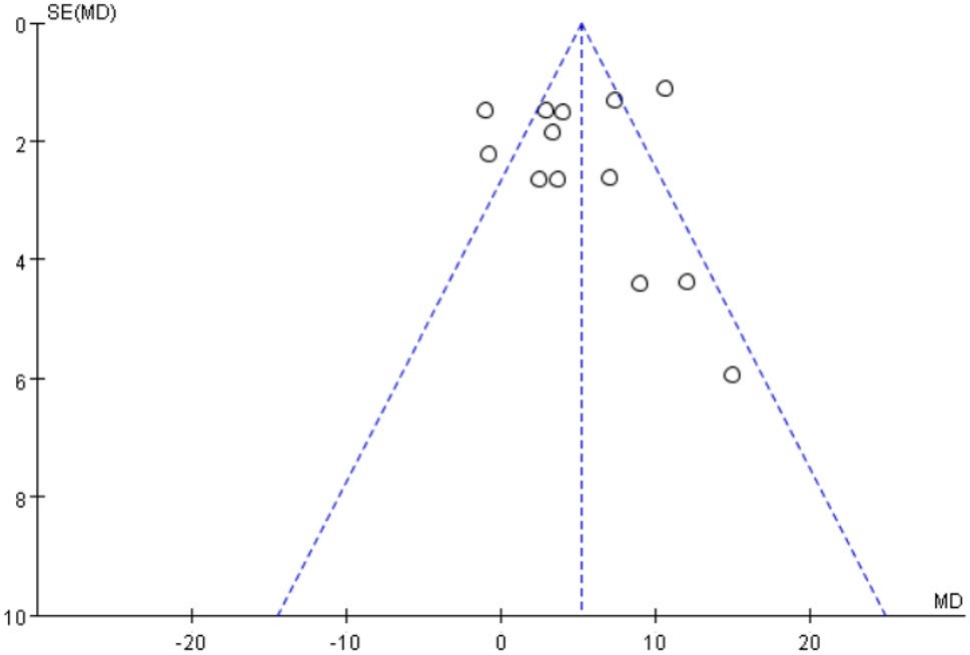
A funnel plot of Lysholm scores. An asymmetry was exhibited in the funnel plot, which reflected the publication bias

Two RCTs showed a higher risk of bias in participant blinding (Fig. 2). All observational studies had an NOS score of 7 or higher, indicating an acceptable methodological quality (Fig. 3).

**Fig. 4:**
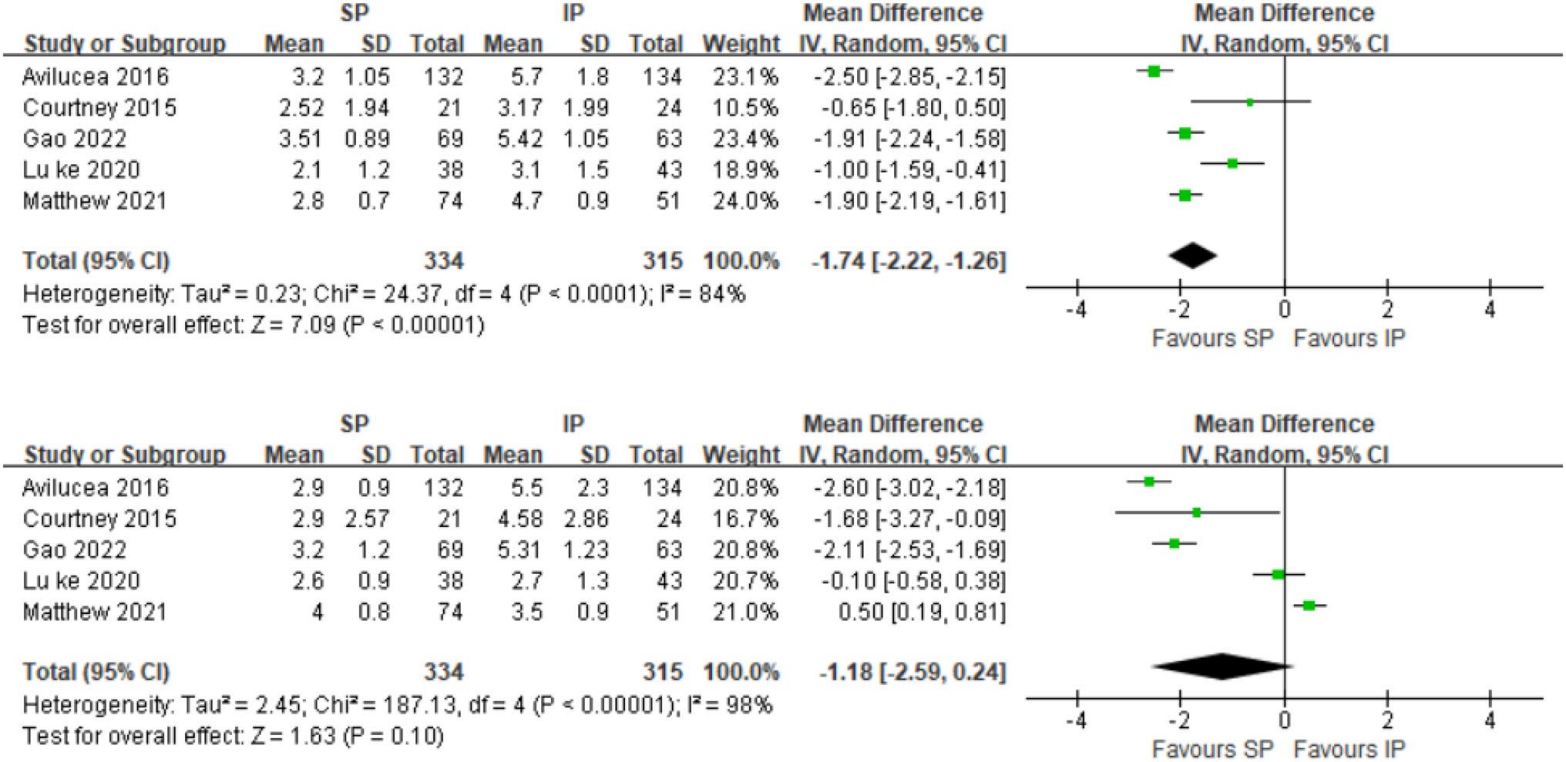
Forest plot for coronal and sagittal plane degrees alignment (°); *SP* suprapatellar; *IP* infrapatellar; *MD* mean difference; *SD* standard deviation; *IV* inverse variance analysis; *CI* confidence interval; *SMD* standard mean difference; *CI* confidence interval

**Fig. 5:**
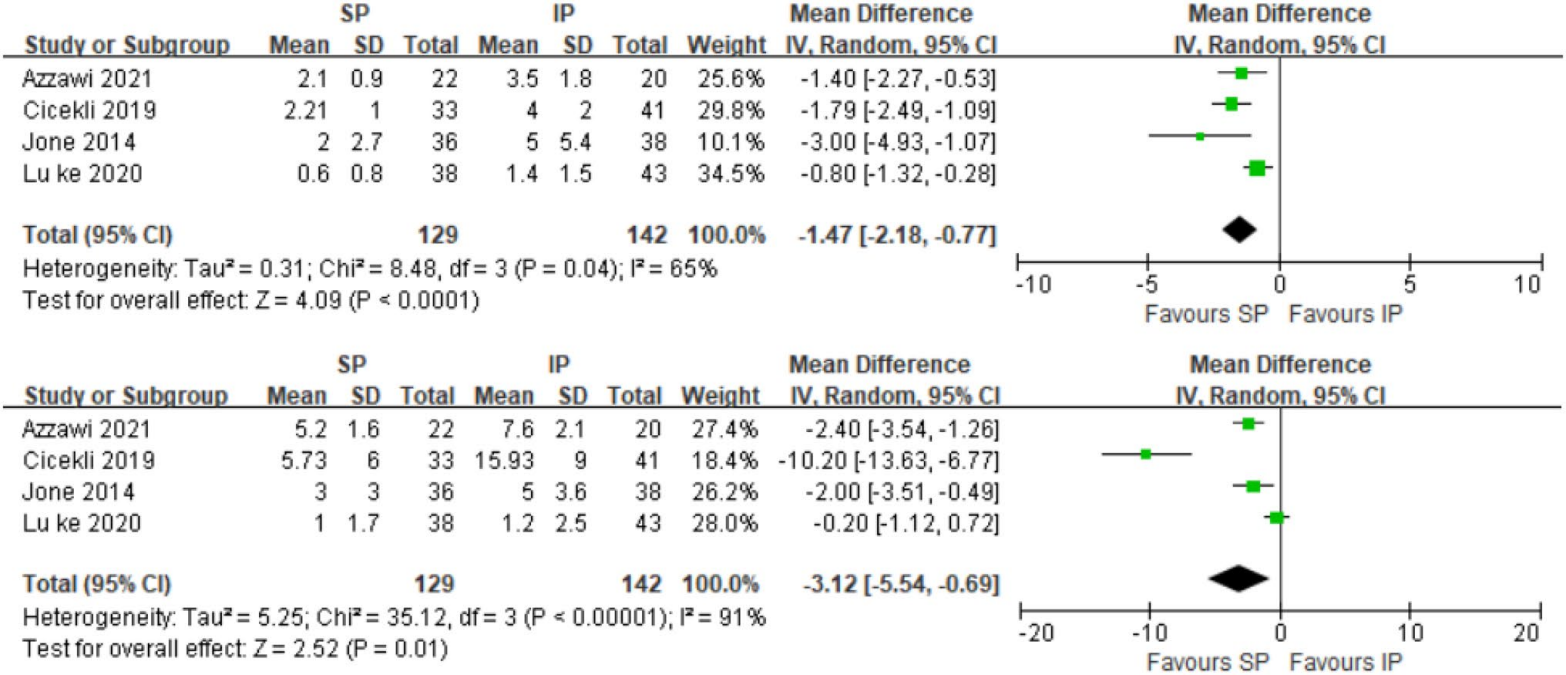
Forest plot for coronal and sagittal plane distance alignment (mm); *SP* suprapatellar; *IP* infrapatellar; *MD* mean difference; *SD* standard deviation; *IV* inverse variance analysis; *CI* confidence interval; *SMD* standard mean difference

**Fig. 6:**
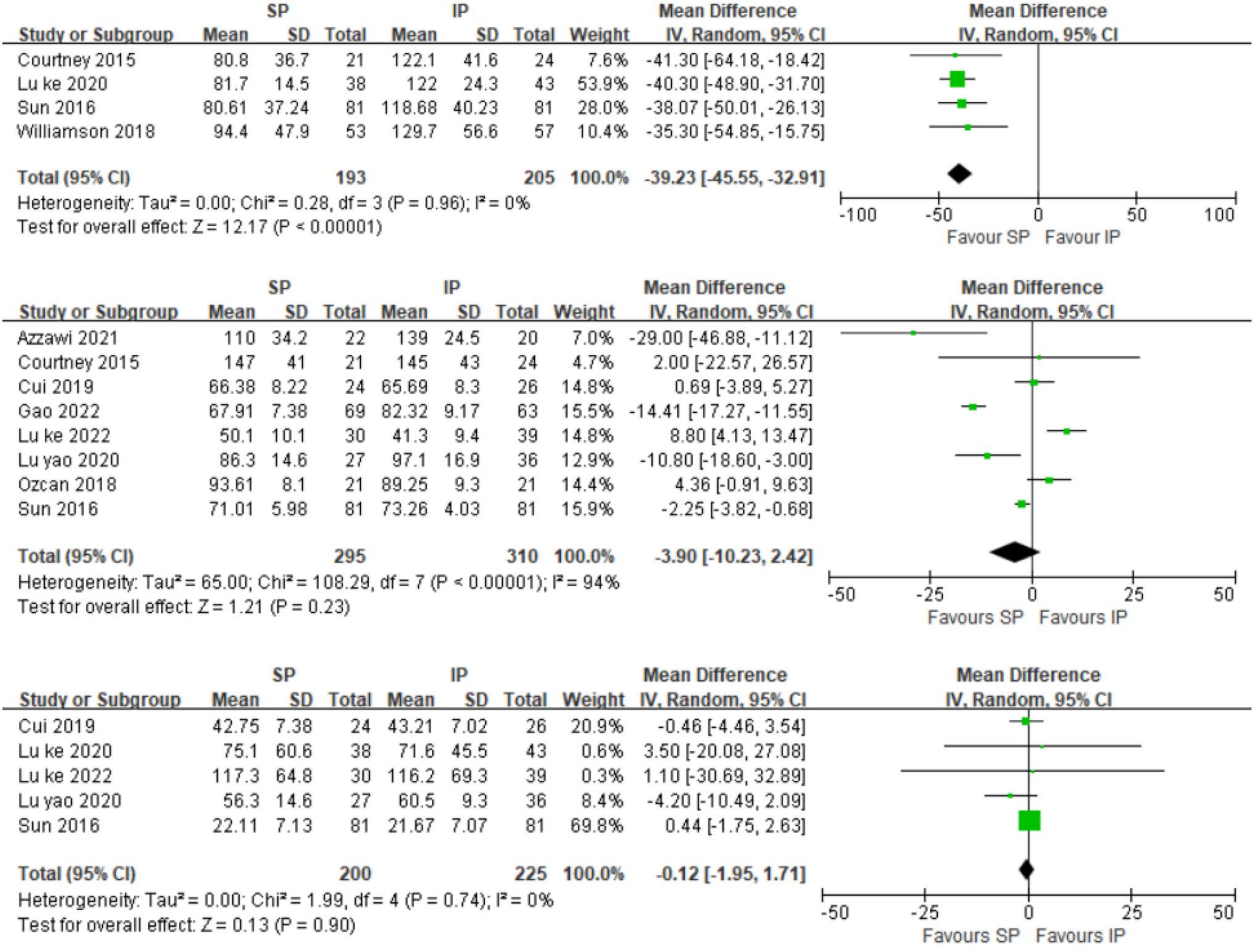
Forest plot for fluoroscopy time (s)、operative time (min) and intraoperative blood loss (ml); *SP* suprapatellar; *IP* infrapatellar; *MD* mean difference; *SD* standard deviation; *IV* inverse variance analysis; *CI* confidence interval; *SMD* standard mean difference

**Fig. 7:**
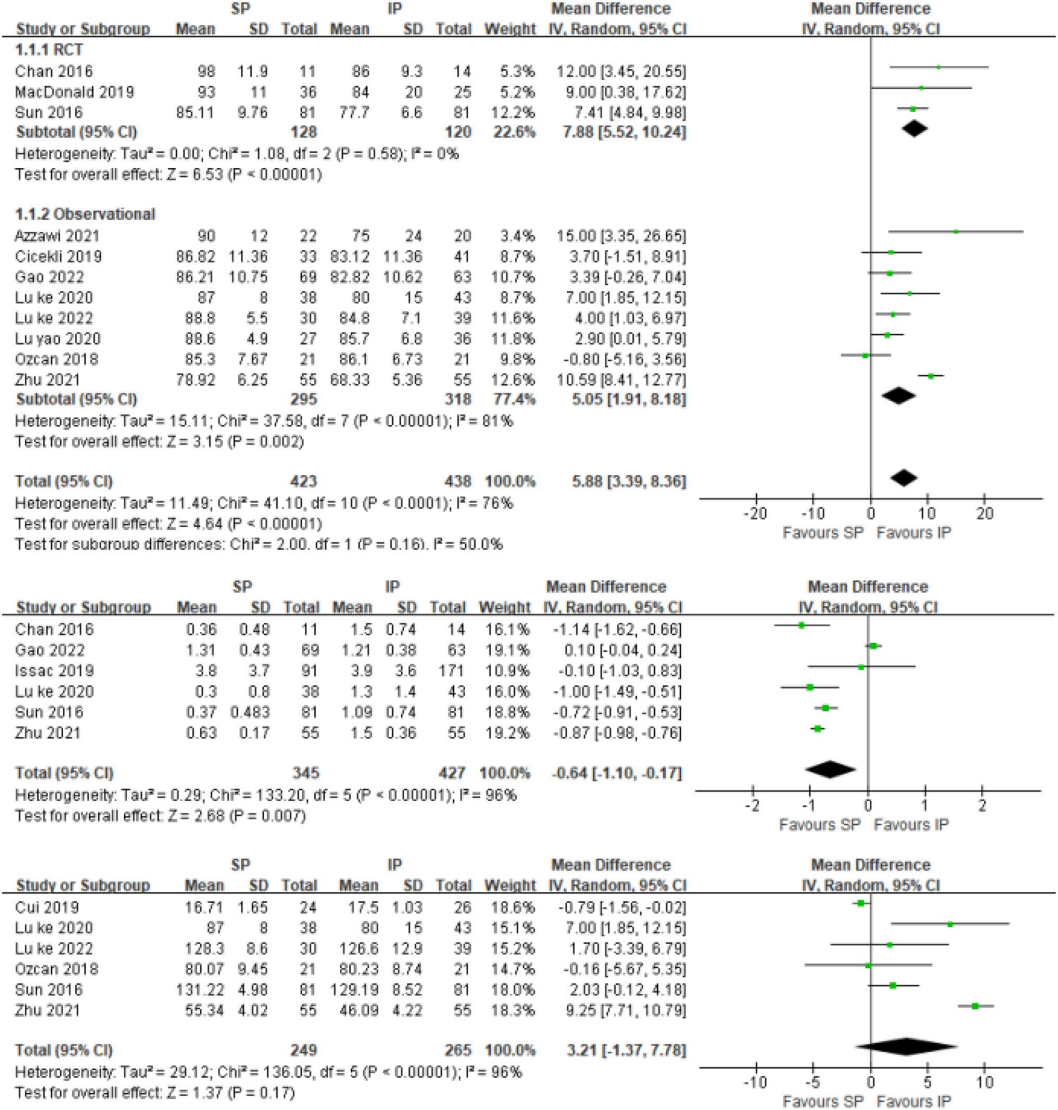
Forest plot for Lysholm knee score、*VAS* pain score and range of motion; *SP* suprapatellar; *IP* infrapatellar; *MD* mean difference; *SD* standard deviation; *IV* inverse variance analysis; *CI* confidence interval; *SMD* standard mean difference

### Alignment

Five studies [20, 21, 41–43] reported the angular alignment and the difference of the angle was significant between the two groups in the coronal plane (SMD =  – 1.74[ – 2.22, – 1.26]; *p* < 0.00001; *I*^2^ = 84%), while it was not significant in the sagittal plane (SMD =  – 1.18[ – 2.59,0.24]; *p* < 0.10; *I*^2^ = 98%) (Fig. 4).

Five studies [23, 25, 29, 33, 43] reported the distance alignment, and one paper was excluded from this meta-analysis because it reported only the median of the quartile spacing, while Anderson et al.[23] found better distance alignment in the coronal plane in their study. Both the differences in the distance between the two groups in the coronal plane (SMD =  – 1.47[ – 2.18, – 0.77]; *p* = 0.04; *I*^2^ = 65%) and sagittal plane (SMD =  – 3.12[ – 5.54, – 0.69]; *p* < 0.01; *I*^2^ = 91%) were significant (Fig. 5) (Table 2).

**Table 2:** Summary results of meta-analysis

Study	Year	Country	Study design	Sample size		Mean age	Male(%)	Open fracture %	Fracture location (proximal/mid/distal)	Follow-up (months)		NOS		
				Total	SP/IP	SP/IP	SP/IP	SP/IP	SP/IP	SP/IP	Selection	Comparability	Outcome	Score
Avilucea [20]	2016	USA	Observational	266	132/134	33.6/35.4	66/56	21/23	(0/0/132) (0/0/134)	NO	***	**	**	7
Courtney [21]	2015	USA	Observational	45	21/24	38.5/37.6	71/46	0/0	(3/10/8) (0/14/10)	11.8/25.2	***	**	***	8
Lu Yao [22]	2020	CN	Observational	63	27/36	43/41	41/56	29/23	(0/0/27) (0/0/36)	23.2/24.3	***	**	***	8
Anderson [23]	2019	UK	Observational	200	95/105	45/35	82.1/78.1	44.2/42.9	NR	NR	***	**	**	7
MacDonald [24]	2019	UK	RCT	95	53/42	42.4/37.6	62/62	15/17	NR	12/12	–	–	–	–
Jones [25]	2014	UK	Observational	76	36/38	40/39	28/42	18/8	(0/26/0) (0/34/0)	22/28	***	**	***	8
Sun [26]	2016	CN	RCT	162	81/81	47/46	82/80	NR	(16/14/51) (17/15/49)	24/24	–	–	–	–
Willianmson [27]	2018	UK	Observational	90	53/37	NR	NR	NR	NR	NR	***	**	***	8
Chan [28]	2016	USA	RCT	25	11/14	40/43	55/71	9/14	(0/10/1) (0/14/0)	15/15	–	–	–	–
Azzawi [29]	2021	UK	Observational	42	22/20	38/32	20/37.5	15/12.5	(0/22/0) (0/20/0)	6/6	***	**	***	8
Zamorano [30]	2022	Spain	Observational	52	22/30	NR	NR	32/23	(0/22/0) (0/30/0)	NR	***	**	**	7
Zhu [31]	2021	CN	Observational	110	55/55	32/33	51/55	NR	(0/55/0) (0/55/0)	6/6	***	**	***	8
Cui [32]	2019	CN	Observational	50	24/26	41/44	67/12	0/0	(0/24/0) (0/26/0)	24/23	***	**	***	8
Çiçekli [33]	2019	Turkey	Observational	74	33/41	43/44	75/73	30/19	(4/22/7) (4/21/16)	29/30	***	**	***	8
Llano [34]	2022	Italy	Observational	80	44/36	48/46	NR	NR	NR	12/12	***	**	***	8
Ozcan [38]	2018	Germany	Observational	58	21/37	31/33.4	90/70	0/0	NR	16/32.5	**	**	***	7
Isaac [39]	2019	Canada	Observational	262	91/171	43.9/40.1	76/64	NR	NR	42.2/50.4	***	**	***	8
Lu ke [40]	2022	CN	Observational	69	30/39	33/35	40/43	NR	(0/30/0) (0/39/0)	6	***	**	***	8
Gao [41]	2022	CN	Observational	132	69/63	46/44	71/60	NR	(0/0/69) (0/0/63)	14	***	**	***	8
Matthew [42]	2021	UK	Observational	125	74/51	41/39	77/75	60/35	(0/0/74) (0/0/51)	NR	***	**	**	7
Lu ke [43]	2020	CN	Observational	81	38/43	37.3/38	61/53	29/23	(0/38/0) (0/43/0)	23.2/24.3	***	**	***	8
Bakhsh [36]	2016	USA	Observational	102	34/68	44/46	58/51	35/38	NR	31/63	**	***	***	8
Ryan [37]	2014	USA	Observational	185	84/101	NR	NR	NR	(50/0/34) (0/101/0)	28	***	***	***	8

*MD* mean difference

*，**，or *** means the Newcastle–Ottawa Scale (NOS) for a systematic studies star rating the more, the better

Overall in terms of alignment, the superiority of the SP over the IP is reflected in the distance alignment in the coronal and sagittal planes, as well as the angular alignment in the coronal plane. It is noteworthy that the included sample almost had no proximal tibial fractures.

### Fluoroscopy time (s)

Six studies [21, 26, 27, 34, 41, 43] reported fluoroscopy exposure during the procedure. Among them, Gao [41] only reported the fluoroscopy number and not time, and Llano [34] only reported the interquartile range of fluoroscopy time. Thus, their studies were excluded from this meta-analysis. Fluoroscopy was used for a significantly shorter amount of time in the SP group (SMD =  – 39.23[ – 45.55, – 32.91]; *p* < 0.00001; *I*^2^ = 0%). Even so, in the two studies that were excluded, the number of fluoroscopies was significantly less in the SP (14.10 ± 2.51) than in the IP group (19.61 ± 3.12) with statistical difference (*p* < 0.001) [41]. The mean time of use of fluoroscopy was 94,5 s in the SP group compared with 204,5 s in the IP group. There was a significant difference (*p* < 0.001) [34] (Fig. 6).

### Operation time (s)

Eight studies [21, 22, 26, 29, 32, 38, 40, 41] reported the operation time. A random-effects model was used. The difference between the two groups in terms of operation time was not significant (SMD =  – 3.90[ – 10.23,2.42]; *p* = 0.23; *I*^2^ = 94%).

### Intraoperative blood loss (mL)

Five studies [22, 26, 32, 40, 43] reported the intraoperative blood loss. A fixed-effects model was used. The difference between the two groups in terms of intraoperative blood loss was not significant (SMD = -0.12[-0.95,1.71]; p = 0.09; I^2^ = 0%).

### Lysholm knee score

Eleven studies [22, 24, 26, 28, 29, 31, 33, 38, 40, 41, 43] reported the Lysholm knee score. A random-effects model was used. In the result of a 6-month follow-up including 851 patients (SP 423, IP 438), there was significant difference between the two groups in terms of Lysholm knee score (SMD = 5.88[3.39, 8.36]; *p* < 0.00001; *I*^2^ = 76%). In the subgroup analysis, the RCT studies showed that a higher Lysholm knee score in the IP group with homogeneity detected (SMD = 7.88[5.52, 10.24]; *p* < 0.00001; *I*^2^ = 0%) (Fig. 7).

### VAS pain score

Six studies [26, 28, 31, 39, 41, 43] reported the anterior knee VAS pain score, and it was not connected with the site of the approach used. A random-effects model was used. There was significant difference between the two groups in terms of VAS pain score. (SMD =  – 0.64[ – 1.10, – 0.17]; *p* = 0.007; *I*^2^ = 96%). We discovered that Gao et al.[41] had a significant impact on heterogeneity by analyzing six studies of sensitivity, After removing the study, the results showed low heterogeneity in the remaining five studies. After exclusion, there was still significant difference between the two groups in terms of VAS pain score. (SMD =  – 0.84[ – 0.93, – 0.76]; *p* < 0.00001; *I*^2^ = 35%). After careful analysis of the literature and the selection criteria, we still did not find the exact source of heterogeneity and decided to keep it in this meta-analysis considering the relevant quality of the study.

### Range of motion

Six studies [26, 31, 32, 38, 40, 43] reported the range of motion. A random-effects model was used. There was no significant difference between the two groups in terms of range of motion (SMD = 3.21[ – 1.37,7.78]; *p* = 0.17; *I*^2^ = 96%).

## Discussion

We included more and newer studies [22, 29–31, 34, 40–43] for this meta-analysis and systematic review, 20 observational studies and 3 RCTs. According to our latest findings, the SP group performed better in entrance alignment and intraoperative fluoroscopy time, and had significantly superior Lymsholm scores and anterior knee VAS scores to the IP group, while we did not find similar results in terms of operative time and intraoperative blood loss.

The SP approach has attracted a lot of attention from scholars in the intraoperative and postoperative conditions. In two meta-analyses published in 2018, the results showed the superiority of the SP group over the IP group in terms of intraoperative blood loss, ROM, VAS, Lysholm, and fluoroscopy time. The authors acknowledged the need for more other high-quality RCTs to confirm these findings [9–12]. In 2021, Packer [13] found differences between the two approaches in Lysholm scores, fluoroscopy time, and entrance alignment in a meta-analysis including 12 studies, while the differences in complications and intraoperative blood loss were not significant. However, Ponugoti [14] found in his meta-analysis that the two approaches differed only in VAS and Lysholm, while there were no significant differences in operative time, fluoroscopy time, deep infection rate, bone nonunion, or secondary procedures. In addition to pain, Lysholm's ratings include instability, thigh muscle atrophy, swelling, and lameness. Despite the correlation between Lysholm and VAS, due to the higher level of heterogeneity resulting from variability in follow-up time, the author stated that their long-term results still need to be confirmed and evaluated in further studies. Sepehri [15] found in a recent meta-analysis that the SP group performed better in Lysholm and intraoperative fluoroscopy time, but showed no such differences in operative time.

The alignment of the entry point is a parameter that is often overlooked by researchers, yet it is equally influenced by the approach. First, mechanically, since the SP approach avoids patellar obstruction, the inserted guide pin easily finds a satisfactory angle while providing a narrow safe entry point for the intramedullary nail to ensure a satisfactory distance deviation [25]; in fluoroscopic slice position, the entry point of the sagittal plane can be clearly confirmed to be located at the junction between the anterior cortex and the articular surface in the tibial plane [43] and therefore did not show any correlation with the two entry points. This may explain the better performance of the entrance point in the coronal plane in terms of accuracy.

In addition, Anderson [23] found that in terms of distance, the accuracy of the entry point in the coronal plane was significantly better than in the sagittal plane, which was similar to our analysis. In cadaveric studies, Franke [17] found that when using the SP approach in the procedure, a more parallel insertion angle to the longitudinal axis was obtained. Thus, both the risk of iatrogenic fracture of the posterior cortex and implant protrusion can be reduced. Therefore, it is reasonable to believe that the suprapatellar approach can help the surgeon to obtain better accuracy in angles and distances in the coronal plane.

According to the accuracy of the achieved reposition, Avilucea [20] reported primary angular disorders in 35 (26.1%) patients in the SP group and 5(3.8%) patients in the SP group (*p* < 0.0001). For valgus and recurvatum, SP performed better than IP. Moreover, Matthew [42] reported that 11 (15%) patients in the SP group had alignment of > 5 degrees from ideal, compared to 17 (33%) patients in the IP group. Gao [41] reported that 3 (4.3%) patients in the SP group and 15 (23.8%) patients in the IP group had poor fracture reduction. Although this seems to indicate that SP has better reduction results, the data are almost exclusively from distal tibial fractures, and the difference between fracture reduction in the middle and proximal tibia remains to be studied more.

Both Chan [28] and Courtney [21] reported one case requiring return to the operating room for exchange intramedullary nailing due to nonunion in the SP group. MacDonald [24] reported 11 further procedure cases in the SP group, in which 6 involved removal of distal locking screws and 5 involved removal of proximal locking screws. These number are three and one in the IP group. Sun [26] reported seven cases of implant loosening, 4(4%) in the SP and three (5%) in IP; they all led to bone nonunion and IMN revision. However, there were no significant differences in the major complication rate that induced revision or implant removal between the two groups. None of our current study included skin incision infections and deep infections, as these are influenced by multiple factors. Also, open fractures were not included in our study because of the high incidence of sepsis [46]. Therefore, the available findings suggest that although the SP group outperformed the IP group in terms of reposition accuracy and remaining malposition, the superiority in the subsequent need for revision may be challenged.

In the analysis of the fluoroscopy time, the SP group took less time than the IP group and the statistical results were discrepant. In the infrapatellar approach, the knee is placed in a hyper-flexed position on the fluoroscopic triangle device, whereas in the suprapatellar approach the semi-extended position of the knee (15–20°) not only facilitates the repositioning, but also allows for a shorter and easier fluoroscopy condition [6]. Moreover, the fluoroscopy time required by itself is substantially reduced due to the easier positioning of the intramedullary nail entry point under the SP approach. Correspondingly, the reduction in fluoroscopy time also shortens the operative time. It was worth mentioning that Wilianmson [7] innovatively focused on the data of radiation exposure (cGY/cm^2^), which was significantly lower in the SP group than in the IP group. Azzawi [29] stated the same conclusion in his study. These suggest that the change in approach will benefit both the surgeons and the patients. In the study of Gao [41], the mean number of fluoroscopy procedures (n) in the SP group was 14.10, significantly lower than that of the IP group at 19.61. Almost all of the studies that included fluoroscopy condition favored the SP group. Therefore, we have reason to believe that fluoroscopy time(s), fluoroscopy number (n), and radiation exposure (cGY/cm^2^) were significantly lower in the SP group than in the IP group.

There are more clinical evaluations of knee function, and due to limitations in literature screening, we only included in our analysis the Lysholm score [8]. This meta-analysis showed that between the two groups, the SP group had a better Lysholm score. Because pain conditions were also included in the Lysholm score, it had some association with the VAS pain score. The outcome of both groups may depend on intraoperative damage to the patellofemoral joint, saphenous nerve, intramedullary nail protrusion, and local inflammation, although the specific device maximally protects the patellofemoral cartilage and talus [10]. Gelbke [9] showed that SP nail insertion contact pressure was high on the patellofemoral joint, but still lower than the threshold of chondrocyte damage. In addition, Leliveld [35] found that the infrapatellar approach may damage the infrapatellar nerve, thereby causing postoperative knee pain and numbness in patients. Katsoulis [4] reported a 47% rate of knee pain after intramedullary nail fixation; however, there is a lack of data to support whether the SP approach will reduce this.

Most of the Lysholm scores in this meta-analysis were from patients 6 months postoperatively. Meanwhile, at the 12-month follow-up, several studies [22, 28, 41] reported the total knee Lysholm scores were comparable between the two groups. Nevertheless, MacDonald [24] reported significant improvements in SP compared to IP at only 1 year. In addition, Sun [26] found tha Lysholm scores were higher in the SP group at both 6 and 24 months after surgery. At about 30 months of follow-up, Çiçekli [33] did not find a significant difference in Lysholm scores between the two groups. These may suggest that the two approaches are fraught with uncertainty in the long-term prognosis of Lysholm scores, even so, we are still confident in the results of this meta-analysis that they differed significantly at least within 6 months after surgery.

Postoperative anterior knee pain was always unavoidable [18], and the sources of pain include implant protrusion, changed biomechanics. and the injury of intra-articular structures, patellar tendon, fat pad, and infrapatellar branch of the saphenous nerve [19]. Theoretically, the change in approach could effectively circumvent some of these factors. For example, in the suprapatellar approach, precise insertion points can avoid damage to the knee structure. A more parallel insertion angle to the longitudinal axis can avoid implant protrusion and infrapatellar nerve injury. These may explain the association between higher insertion point accuracy and lower VAS scores.

Likewise, most of the VAS scores in this meta-analysis were also from patients 6 months postoperatively. In the long-term prognosis, Sun [26] showed significant difference between the two groups at 12 and 24 months postoperatively; he also reported the conditions at 1and 3 months, but the outcomes were not significant. Lu [43] reported better performance in the SP group at 12 months, though it contradicted the findings of some studies [28, 30, 41].

Although some former studies [25, 28, 36, 37] did not find any difference in VAS scores between SP and IP, more data from the combined meta-analysis still gives us confidence in the results. Considering the high heterogeneity for some results of this meta-analysis, it should be treated with caution, which also means that we need more high-quality studies.

Although our meta-analysis incorporated more recent studies, explored most of the relevant questions, and reached the latest conclusions in the two groups, several potential limitations should be noted: (1) we were unable to include more RCTs in the literature due to the specificity of the study; (2) in the face of high heterogeneity, we were not able to explore their sources; (3) the prognosis of short-term follow-up within 6 months as well as infection, deformity,and bone nonunion was not comprehensive due to literature limitations.

## Conclusion

The results of our meta-analysis showed that the SP approach was significantly better than the IP approach in angle and distance entrance accuracy of coronal plane, angle entrance accuracy of sthe agittal plane, fluoroscopy time, Lysholm score, and VAS pain score. There were no significant differences in the sagittal angle accuracy, operative time, intraoperative blood loss, and ROM score. Moreover, the specificity of the SP approach may be more suitable for young surgeons to learn, and in terms of safety, no significant patellar injury was observed in patients undergoing the SP approach.

## Data Availability

The authors declare that all the data supporting the findings of this study are available within the article and its supplementary information files.
